# Carbon Monoxide Fumigation Improved the Quality, Nutrients, and Antioxidant Activities of Postharvest Peach

**DOI:** 10.1155/2014/834150

**Published:** 2014-11-17

**Authors:** Shaoying Zhang, Ying Li, Fei Pei

**Affiliations:** College of Food Science, Shanxi Normal University, Linfen 041004, China

## Abstract

Peaches (*Prunus persica* cv. Yanhong) were fumigated with carbon monoxide (CO) at 0, 0.5, 5, 10, and 20 *μ*mol/L for 2 hours. The result showed that low concentration CO (0.5–10 *μ*mol/L) might delay the decrease of firmness and titrable acid content, restrain the increase of decay incidence, and postpone the variation of soluble solids content, but treating peaches with high concentration CO (20 *μ*mol/L) demonstrated adverse effects. Further research exhibited that exogenous CO could induce the phenylalnine ammonialyase activity, maintain nutrient contents such as Vitamin C, total flavonoid, and polyphenol, and enhance antioxidant activity according to reducing power and 2,2-diphenyl-1-(2,4,6-trinitrophenyl) hydrazyl radical scavenging activity. Treating peaches with appropriate concentration CO was beneficial to the quality, nutrients, and antioxidant activity of postharvest peaches during storage time. Therefore, CO fumigation might probably become a novel method to preserve postharvest peach and other fruits in the future.

## 1. Introduction

Peach is one of the most well-liked fruits in the world because of their flavor, dietary value, attractive color, and medicinal worth. It is enriched with ascorbic acid, flavonoid, and phenolic compounds, which were considered prime sources for antioxidants [1, 2]. However, peaches have a very short shelf life because they are highly susceptible to pathogenic infection and physiological deterioration during storage time under ambient temperature. Cold storage remains the main method to slow the product deterioration in terms of consumer perception and nutritional value. However, low temperature results in chilling injury symptoms in some peach cultivars during or after cold storage [3, 4]. Chemical treatments have been used to prevent insect attack and prolong shelf life of postharvest peach. However, the use of chemicals has been minimized for food safety and environmental reasons. Many physical methods including modified atmosphere packaging, heat, and UV-C pretreatments are being extensively studied as substitutes for current chemical methods in the commercial applications of peach [5–8].

Carbon monoxide (CO) is a simple diatomic gas molecule with low water solubility. It easily combines hemoglobin, thus delaying oxygen transport and leading to death of organisms. So in the past, it was considered a toxic gas to environment and biology. However, recent researchers found that, similar to NO and H_2_S, CO might serve as a gaseous signal molecule to involve in stomata close regulated by plant guard cell and the formation of lateral root. Meanwhile, CO may relieve plant tissue oxidation damage caused by a variety of abiotic stresses such as heavy metal, salt, and active oxygen donor [9–13]. It has been reported that exogenous CO donor treatment might postpone the senescence of cut flower through regulating active oxygen metabolism and inhibiting lipid peroxidation [14]. Our previous study found that exogenous CO treatment could restrain the browning of fresh cut lotus root and prolong the shelf life of postharvest jujube [15, 16].

At present, there are few reports about the effect of CO on plant senescence and fruit preservation, and the physiological mechanism of CO on plant senescence stress is still unclear. In this work, the postharvest peaches were fumigated with exogenous CO gas at different concentrations, and the presentation quality, nutrients, and antioxidant activity of peaches were determined periodically during the storage time. This research aims to investigate the effect of CO treatment on postharvest peach and try to explore a novel method for fresh peach preservation.

## 2. Materials and Methods

### 2.1. Materials

Peaches (*Prunus persica* cv. Yanhong) were purchased from an orchard situated in Yaodu District, Linfen City, Shanxi Province. The fruits were picked at commercial mature stage. Peaches with uniform shape, size, and color, as well as no insect pest and mechanical damage, were selected and quickly transported to the laboratory of Shanxi Normal University in open cartons.

CO gas with a purity of 99.99% was purchased from Beijing Huaneng Special Gases Co., Ltd. (Beijing, China). Oxalic acid, gallic acid, rutin, potassium ferricyanide, trichloroacetic acid, and sodium hydroxide (analytical grade) were purchased from Sinopharm Chemical Reagent Co., Ltd. (Shanghai, China). Analytical-grade 1,1-diphenyl-2-picrylhydrazyl radical, 2,2-diphenyl-1-(2,4,6-trinitrophenyl) hydrazyl, and 2,6-dichloroindophenol were purchased from Sigma (USA). Other reagents of analytical grade were purchased from Alfa Aesar Company (Tianjin, China).

### 2.2. Fruit Treatment

The peaches were fumigated with CO gas at different concentrations (0.5, 5, 10, or 20 *μ*mol/L) for 2 hours under ambient temperature, respectively. Detail was as follows. About 10 kg of peaches were placed into a glass container, and then the container was sealed with lid. The diameter and height of used container are 40 cm. CO gas was injected into the glass container through the port of lid. The peaches in the container were fumigated with CO for 2 hours. Peaches that had not been fumigated with CO gas were also sealed in glass container for 2 hours, serving as control sample. After treatment, all samples were then placed in plastic bags and stored under ambient temperature with 90% of relative humidity. Each sample was about 180 peaches and the relevant parameters for the analyses of peaches were measured periodically.

### 2.3. Determination of Firmness and Decay Incidence

Firmness was determined using a fruit digital sclerometer 8 mm in diameter for the head (GY-4, Chendu Bsida Instrument Co., Ltd., Chendu, China). For each fruit, two readings were taken in the equatorial region of the fruit after the skin was removed. The firmness of six fruits during storage was regularly measured and expressed as kg/cm^2^ [8].

Decay incidence was assayed as Santana et al. [17]. It was determined visually in the fruits from three trays and rated as 0 = absent; 1 = very slight (1% ≤ surface ≤ 10%); 2 = slight (10% ≤ surface ≤ 25%); 3 = moderate (25% ≤ surface ≤ 50%); 4 = severe (50% ≤ surface ≤ 100%). Healthy fruit were those showing no signs of decay. The decay incidence for the treatment unit was calculated as follows: decay incidence = [(Σ rank × quantity)/(4 ×  *N*)] × 100%. *N* is the total number of fruits.

### 2.4. Determination of Soluble Solids Content and Titratable Acidity

Soluble solids content and titratable acidity were assayed according to the method of Mignani with modifications [18]. Tissues (50 g) from ten fruits were homogenised and then centrifuged at 8000 ×g for 20 min using an Eppendorf 5417R centrifuge (Germany). The supernatant was collected to measure soluble solids content (Brix) using a refractometer (WYT-II, Qingyang Optical Instrument Co., Ltd., Chendu, China).

Titratable acidity expressed as citric acid on a fresh weight basis was determined by titration with 0.1 mol/L NaOH to pH 8.2. The pH of the supernatant was measured using a pH meter (PHS-3TC, Shanghai Leici Instrument Inc., Shanghai, China).

### 2.5. Determination of Phenylalanine Ammonialyase (PAL) Activity

PAL activity was determined as per the method of Hussain et al. [19] with modification. 5 g of sample from six fruits was homogenized in 5 mL of borate buffer (0.05 M, pH 8.0) containing 5 mM *β*-mercaptoethanol and 1 mM EDTA. Homogenate was centrifuged at 8000 ×g for 15 min at 4°C. The supernatant was collected for enzyme assay. About 1.0 mL of enzyme extract was incubated with an assay medium containing 2 mL of 200 mmol/L sodium borate buffer (pH 8.0), 1 mL of distilled water, and 1 mL of 50 mmol/L 1-phenylalanine as substrate at 30°C for 1 h. The reaction was terminated by adding 0.2 mL of 6 mol L^−1^ HCl. PAL activity was measured by the change in absorbance at 290 nm. One unit was defined as the change 0.01 absorbance at 290 nm per h.

### 2.6. Determination of Vitamin C, Total Polyphenol, and Total Flavonoid Content

Vitamin C content was measured through 2,6-dichloriondophenol titration [20]. Briefly, tissues (5 g) from 6 fruits were homogenised in 10 mL of 2% oxalic acid solution and then centrifuged at 8000 ×g for 15 min at 4°C. Afterwards, 2 mL of the supernatant was titrated to a permanent pink colour using 0.1% of 2,6-dichlorpphenolindophenol. Vitamin C concentration was calculated according to the titration volume of 2,6-dichloriondophenol and expressed as *μ*g g^−1^ fresh weight.

Total polyphenol content was determined using Folin-Ciocalteu's phenol reagent via spectrophotometric analysis [21]. Tissues (5 g) from 10 fruits were homogenised in 20 mL of 50% aqueous methanol and then centrifuged at 3000 ×g for 20 min. The clear supernatant was collected. An aliquot (1 mL) of a standard solution of gallic acid with 0, 10, 20, 30, 40, and 50 mg/L aqueous methanol or supernatant was added to a 25 mL volumetric flask containing 9 mL of water. Approximately 1 mL of Folin-Ciocalteu's phenol reagent was added to the mixture and then shaken. After 8 min, 2 mL of 7.5% aqueous Na_2_CO_3_ solution was added. The solution was immediately diluted with water to a final volume of 25 mL and thoroughly mixed. After incubation for 30 min at 25°C, the absorbance versus the prepared blanks was read at 765 nm. Total polyphenol content was expressed as *μ*g gallic acid equivalents per g fresh weight.

Total flavonoid content was measured through colourimetric assay [22]. Tissues (5 g) from 10 fruits were homogenised in 20 mL of 80% ethanol and then centrifuged at 3000 ×g for 20 min. The clear supernatant was collected. An aliquot (1 mL) of a standard solution of rutin with different concentrations (0, 10, 20, 30, 40, and 50 mg/L) or supernatant was added to 10 mL volumetric flasks containing 4 mL of water. At the onset of each experiment, 0.4 mL of 5% NaNO_2_ was added to the flask. After 5 min, 0.4 mL of 10% AlCl_3_ was added. After 6 min, 2 mL of 4% NaOH was added. Immediately, the solution was diluted with water to a final volume of 10 mL and thoroughly stirred. The absorbance of the mixture was determined at 510 nm versus the prepared blanks. Total flavonoid content was expressed as *μ*g rutin equivalents per g fresh weight.

### 2.7. Determination of Antioxidant Activity

Total antioxidant activity (TAA) was quantified according to the method of Sayyari et al. [23] with modifications, which enables one to determine TAA due to both hydrophilic and lipophilic compounds in the same extraction. Briefly, for each subsample, tissues (10 g) from 10 fruits were homogenized in 10 mL of 50 mM phosphate buffer, pH 7.8, and 5 mL of ethyl acetate and then centrifuged at 10000 ×g for 15 min at 4°C. The upper fraction was used for TAA due to lipophilic compounds (L-TAA) and the lower for TAA due to hydrophilic compounds (H-TAA). In both cases, TAA was determined using the 2,2-diphenyl-1-(2,4,6-trinitrophenyl) hydrazyl (DPPH) radical scavenging activity and reducing power, respectively.

The reducing power of the fruit samples was determined using the method of Jayaprakasha et al. [24]. A 0.2 mL aliquot of the supernatant was mixed with 2.5 mL of phosphate buffer (0.2 M, pH 6.6) and 2.5 mL of 1% potassium ferricyanide in 10 mL test tubes. The mixtures were incubated for 20 min at 50°C. After incubation, 1 mL of 10% trichloroacetic acid was added to the mixtures, followed by centrifugation at 5000 ×g for 10 min. The upper layer (2.5 mL) was mixed with 2.5 mL of distilled water and 1 mL of 0.1% ferric chloride. The absorbance was measured at 700 nm. The reducing power test was run in triplicate. The increase in absorbance of the reaction mixture indicated the reducing power of the samples. H-RP stands for hydrophilic compounds reducing power, and L-RP stands for lipophilic compounds reducing power.

DPPH radical scavenging capacity was assayed as described by Yang et al. [25] with slight modifications. Briefly, 0.2 mL of the supernatant was added to 3 mL of DPPH (120 *μ*mol/L) in methanol. A spectrophotometer (UV-1100, Shanghai Meipuda Instrument Co., Ltd., Shanghai, China) was used, and the absorbance at 517 nm was after the reaction mixtures were incubated for 1 h at 30°C in the dark. The scavenging rate of DPPH radicals was calculated as scavenging rate (%) = [1 − (*A*_1_ −* A*_*s*_)/*A*_0_] × 100, where* A*_0_ is the absorbance of the control solution (3 mL of phosphate-buffered saline in 3 mL of DPPH solution),* A*_1_ is the absorbance of the supernatant in DPPH solution, and* A*_*s*_, which is used for error correction arising from unequal colour of the sample solutions, is the absorbance of the sample extract solution without DPPH. H-DRSC represents the DPPH radical scavenging capacity of hydrophilic compounds, and L-DRSC represents the DPPH radical scavenging capacity of lipophilic compounds.

### 2.8. Statistical Analysis

Each treatment was repeated three times, and the data were processed by analysis of variance using DPS7.05 statistical software (Refine Information Tech. Co., Ltd., Hangzhou, China). The treatments were compared at *P* = 0.05 using Tukey's test, which indicates the multicomparison value in each case. The data were expressed as mean ± standard deviation (*n* = 3). Pearson correlations were used to determine the relationship among measured variables (physical and physiological responses and antioxidant contents and activity).

## 3. Results and Analysis

### 3.1. Firmness and Decay Incidence

**Figure 1:**
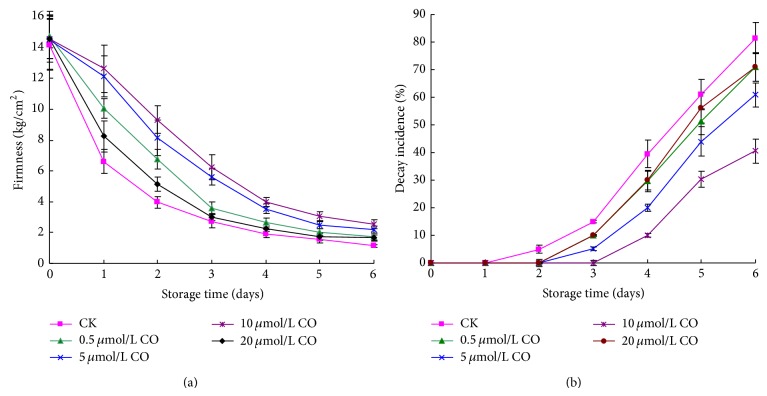
Effect of CO fumigation on the firmness (a) and decay incidence (b) of postharvest peaches. Each point represents the mean value ± SD.

The cultivar peach of Yanhong belongs to melting-flesh fruit and its firmness decreased rapidly with fruit senescence. As shown in Figure 1(a), the firmness of postharvest peach decreased quickly during the first 3 days of storage and then declined slowly from 3 to 6 days. CO fumigation could reduce the firmness decrease of postharvest peaches. When peaches were fumigated with concentration of CO (0.5–10 *μ*mol/L), the decrease of firmness became slow with the CO concentration enhancement. Whereas the peaches were treated with higher concentration CO (20 *μ*mol/L), their firmness decreased quickly. From the first day of storage time, the firmness of peaches fumigated with 5 or 10 *μ*mol/L CO was significantly higher than that of control samples. Particularly the peaches treated with 10 *μ*mol/L CO demonstrated the slowest rate of firmness decline, and its firmness was 2.24 times of control fruit on day 6.

Postharvest peaches easily softened and decayed under ambient temperature. As described in Figure 1(b), peaches began to rot at day 2, and its decay incidence increased rapidly after 3 days. On day 6, the decay incidence reached 81.44%. Fortunately, CO fumigation might restrain the decay of postharvest peaches. During the whole storage time, the decay incidence of all treated samples was lower than that of control fruits. Of all these samples, peaches treated with 10 *μ*mol/L CO showed the lowest increase of decay incidence, and there was significant difference compared to that of control samples (*P* < 0.01). At day 6, the decay incidence of 10 *μ*mol/L CO treated peaches was 40.50%, which was only 49.73% of control samples.

### 3.2. Soluble Solids Content and Titratable Acid

**Figure 2:**
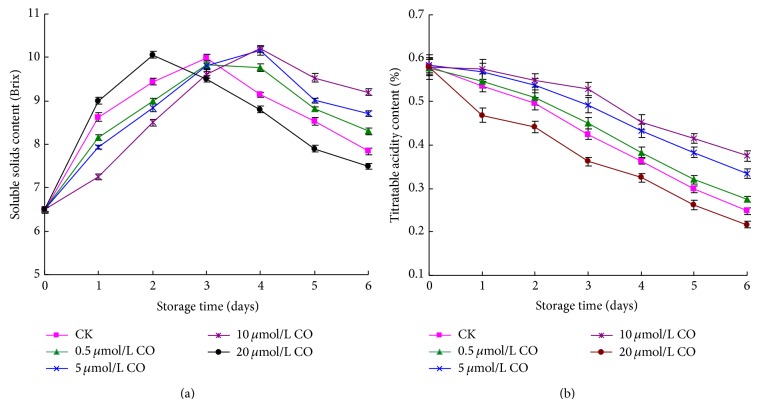
Effect of CO fumigation on soluble solids content (a) and titratable acid (b) of postharvest peaches. Each point represents the mean value ± SD.

As shown in Figure 2(a), the soluble solids content of postharvest peaches first gradually increased and then slowly decreased during the storage time. Both control sample and 0.5 *μ*mol/L CO treated sample appeared to have the maximum value at day 3, and 20 *μ*mol/L CO treated peaches displayed the peak value at 2 day. While 5 or 10 *μ*mol/L CO treated sample exhibited the maximum value on day 4, postponing 1 day compared to that of control sample. From 4 to 6 days, the soluble solids content of 10 *μ*mol/L CO treated fruits decreased the slowest. Therefore, fumigating peaches with 10 *μ*mol/L CO could effectively reduce the decrease of soluble solids content.

The titratable acid of postharvest peaches decreased during storage time (Figure 2(b)). 20 *μ*mol/L CO treated peaches showed the fastest decrease followed by the control sample and 0.5 *μ*mol/L CO treated sample. The titratable acid of 5 or 10 *μ*mol/L CO treated sample decreased the least during storage time, which is higher than that of other samples from 3 to 6 days (*P* < 0.05). At day 6, the titratable acid content of 10 *μ*mol/L CO treated peaches was 72.8% higher than that of control samples.

### 3.3. Vitamin C, Total Flavonoid, and Total Polyphenol Content

**Figure 3:**
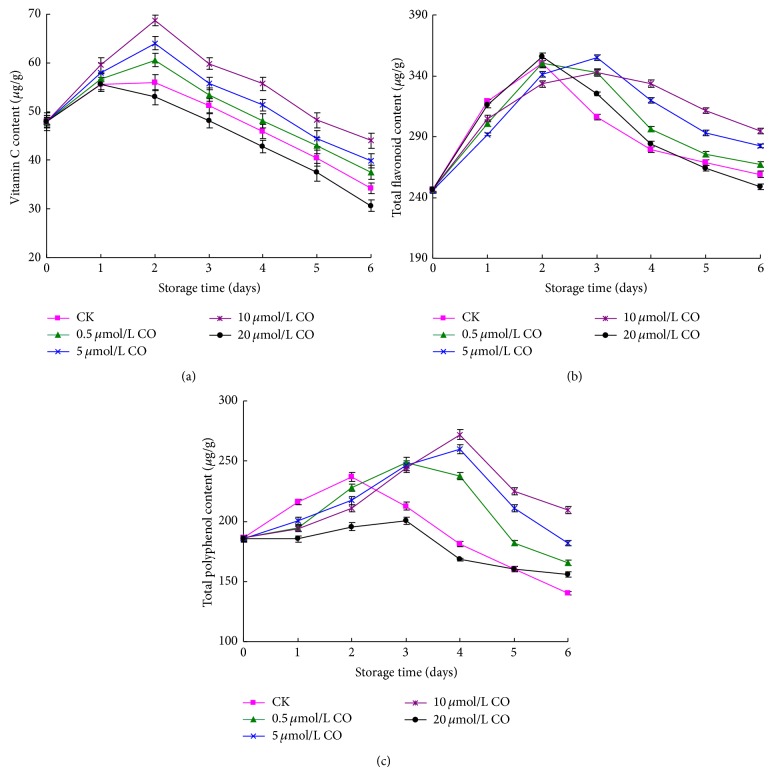
Effect of CO fumigation on vitamin C (a), total flavonoid (b), and total polyphenol (c) content of postharvest peaches. Each point represents the mean value ± SD.

The vitamin C of postharvest peaches showed increasing trend in the first two days and then decreased from 2 to 6 days (Figure 3(a)). The vitamin C content of 20 *μ*mol/L CO treated peaches reached the peak value at day 1 and then decreased. It was the lowest during the storage time compared to control and all other treated samples. Other samples appeared to have the maximum value of Vitamin C at day 2, and the Vitamin C contents of treated samples with 0.5–10 *μ*mol/L CO were higher than those of control samples. Furthermore, the peaches treated with 10 *μ*mol/L CO showed the highest Vitamin C content among all samples during the storage time, and significantly higher than that of control samples (*P* < 0.01).

As described in Figure 3(b), the total flavonoid content of peaches first increased and then decreased during the whole storage time. The peaches treated with 0.5 or 20 *μ*mol/L CO and control sample appeared to have the maximum of total flavonoid content at day 2, and there was no difference among them. 5 or 10 *μ*mol/L CO exhibited peak value of total flavonoid content at day 3, but the total flavonoid content of peaches treated with 10 *μ*mol/L CO was significantly higher than that of other samples from 4 to 6 days (*P* < 0.05). Similar to total flavonoid content, the total polyphenol content firstly increased and then decreased as well (Figure 3(c)). The total polyphenol content of control sample rapidly reached maximum at day 2. 0.5 or 20 *μ*mol/L CO treated peaches appeared to have the peak value of total flavonoid content on day 3, but the total polyphenol content of peaches treated with 20 *μ*mol/L CO was significantly lower than that of other samples. The total polyphenol content of peaches treated with 5 or 10 *μ*mol/L CO increased the slowest, and it reached the maximum at day 4. Obviously, treating peaches with 5 or 10 *μ*mol/L CO might restrain the decrease of total polyphenol content of postharvest peaches.

### 3.4. PAL Activity

**Figure 4:**
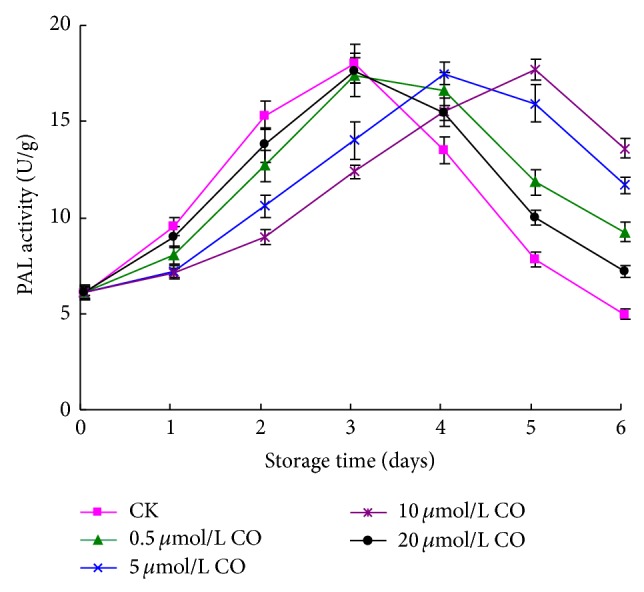
Effect of CO fumigation on PAL activity of postharvest peaches. Each point represents the mean value ± SD.

As shown in Figure 4, the PAL activity of postharvest peaches showed an obvious peak value during storage time. The sample treated with 0.5 or 20 *μ*mol/L CO and control sample appeared to have maximum of PAL activity at day 3, but the rising and falling speed of PAL activity of peaches treated with 0.5 or 20 *μ*mol/L CO was lower than that of control sample. Peaches treated with 5 and 10 *μ*mol/L CO demonstrated peak value of PAL activity at days 4 and 5, and they were postponed at 1 and 2 days, respectively. Apparently, peaches treated with 10 *μ*mol/L CO maintained higher PAL activity during the last storage time.

### 3.5. Antioxidant Activities

**Figure 5:**
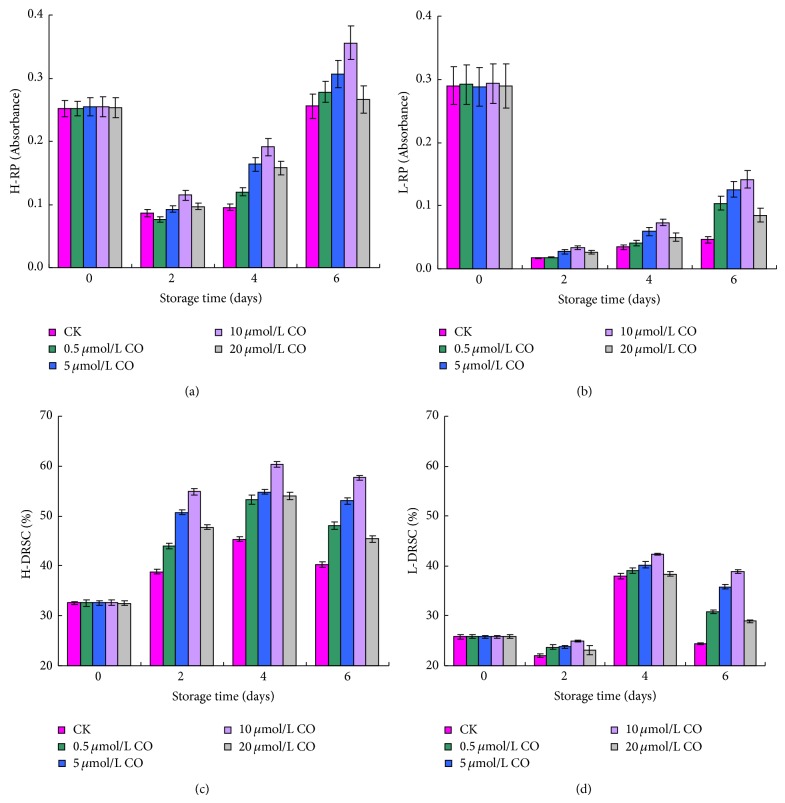
Effect of CO fumigation on H-RP (a), L-RP (b), H-DRSC (c), and L-DRSC (d) of postharvest peaches. Each point represents the mean value ± SD. H-RP stands for hydrophilic compounds reducing power, and L-RP stands for lipophilic compounds reducing power. H-DRSC represents the DPPH radical scavenging capacity of hydrophilic compounds, and L-DRSC represents the DPPH radical scavenging capacity of lipophilic compounds.

The H-RP and L-RP of postharvest peaches all firstly promptly decreased and then gradually increased during the storage time, but there were some differences between them (Figures 5(a) and 5(b)). At 0 day, the H-RP was slightly lower than L-RP. However, it was significantly higher than L-RP during the storage time and was about 2.8–5.6 times compared to L-RP. Moreover, at the end of storage, the H-RP still maintained high level, while the L-RP decreased less 1/6 than that of original level. Compared to that of control sample, the H-RP or L-RP in all CO treated samples was higher. Obviously, exogenous CO fumigation was beneficial to maintaining the reducing power of postharvest peach, and 10 *μ*mol/L CO treatment demonstrated the best effect.

As shown in Figure 5(c), the H-DRSC of postharvest peach began to increase at day 2 and slightly decreased at day 6. All CO treated samples showed significantly higher H-DRSC between 2 and 6 days compared to that of control sample (*P* < 0.05). Of all these samples, peaches treated with 10 *μ*mol/L CO displayed the highest DPPH radical scavenging activity. The L-DRSC of postharvest peach firstly decreased, then increased, and decreased again during the whole storage period (Figure 5(d)). After 2 days, the L-DRSC of control sample was lower than that of CO treated samples. At day 6, 10 *μ*mol/L CO treated sample showed the highest DPPH radical scavenging activity, which was about 1.6 times of control samples. Compared with H-DRSC of postharvest peach, the L-DRSC was markedly lower, yet it could be significantly improved after the peaches were fumigated with CO.

## 4. Discussion

Peach, a respiratory climacteric fruit, is easy to soften and rot under ambient temperature after harvest. Therefore, firmness and decay incidence were important indexes of postharvest peaches. The results showed that the firmness of postharvest peaches rapidly decreased under ambient temperature, from 14.18 kg/cm^2^ at day 0 to 2.74 kg/cm^2^ at day 3. So the peach used in our experiment belonged to the typical cultivar of melting-flesh type [4]. After exogenous CO fumigation, the firmness decrease and decay incidence increase of postharvest peaches were restrained during storage time. Furthermore, in low concentration (0.5–10 *μ*mol/L), the firmness of CO treated fruit decreased slowly and decay incidence increased slowly as well with CO concentration enhancement. However, when CO concentration increased to 20 *μ*mol/L, the firmness of postharvest quickly decreased and accordingly the decay incidence fast increased. As shown in Table 1, there were significant negative correlations between firmness variation and decay incidence of postharvest peaches during storage time. Namely, with firmness decrease, decay incidence gradually increased. The reason was probably that gradual fruit senescence led to the decomposition of intercellular pectin of flesh and cell separations with storage time extension. Thus, peaches softening occurred and the integrity of fruit cell wall structure was destroyed [26]. Afterward, fruit physiological metabolism was disordered and the resistance to exogenous pathogen was gradually lost. As a result, decay incidence of postharvest peaches gradually increased.

Postharvest peach eventually appeared to have some physiological changes owing to respiratory metabolism and ethylene production during senescence process. These changes included flesh softening, starch degradation, flavor variation, and organic acid content decrease [27]. Soluble solids and titratable acid content represented the quality of postharvest peaches, and their ratio was also an important index to reflect the maturity and senescence of postharvest fruit [28]. The soluble solids content of cultivar peach “Yanhong” firstly increased and then decreased during storage period. This reason probably related with starch degradation. The amylase of postharvest peaches catalyzed the starch into soluble sugar and soluble solids content increased accordingly. After storage for some time, the starch became less and the sugar production gradually lessened. Meanwhile, the physiological metabolism of postharvest peaches continued to consume sugar. As a result, the soluble solids content decreased with storage time extension. Our experimental results showed that CO had dual effects. Treating peaches with 0.5–10 *μ*mol/L exogenous CO might inhibit the decrease of titratable acid content, postpone the variation of soluble solids and vitamin C content, and effectively maintain the qualities of postharvest peaches. However, treating peaches with 20 *μ*mol/LCO demonstrated adverse effect. This phenomenon was similar to NO applied in postharvest fruit and vegetable [29].

Peach has good flavor and abundant nutrients, preventing many diseases of mankind induced owing to the accumulation of reactive oxygen compounds in organism [30, 31]. Flavonoid and polyphenol, as main antioxidant substance originating from plant, are important functional phytochemicals of peach fruit. The experimental result showed that CO treating could postpone the flavonoid and polyphenol variation of postharvest peach and maintain them in higher level. The reason was probably that CO treating induced the PAL activity of peach flesh. The correlation coefficients of PAL activity relating with total flavonoid, total polyphenol, and total flavonoid + total polyphenol, were 0.48, 0.72, and 0.64, respectively. El-Samahy et al. [32] also considered that the polyphenol increase of flesh related with PAL activity. PAL is the key enzyme for the metabolism of polyphenol. It catalyzes the deamination of L-phenylalanine to yield ammonia and transcinnamic acid, from which phenolic compounds including flavonoid, phenolic acid, and anthocyanin, are produced [19]. Therefore, CO probably increased the flavonoid and polyphenol content through heightening PAL activity, maintaining the physiological function of postharvest peach. Many polyphenol compounds are known to show antioxidant activity. These have hydrogen with activity that causes the hydrogen exchange reaction with free radical and structure of resonance stabilized [33]. Research suggested that the antioxidant activity of postharvest peaches closely related with flavonoid and polyphenol content [34]. As shown in Table 1, the flavonoid content of postharvest peaches significantly correlated H-DRSC (*r* = 0.89) and moderately correlated T-DRSC (*r* = 0.70). Polyphenol content closely correlated H-DRSC, L-DRSC, and T-DRSC, and these correlation coefficients were 0.72, 0.73, and 0.81, respectively. In addition, the content of total flavonoid + total polyphenol also significantly positively correlated with H-DRSC and T-DRSC (*r* = 0.87 and *r* = 0.81). These results were similar to Kim et al. [27], who treated peach with gamma irradiation. Obviously, CO treating could increase the flavonoid and polyphenol content of postharvest peach, and accordingly the antioxidant activities of flesh were enhanced in terms of DPPH radical scavenging capacity. The experimental result also exhibited that the content of total flavonoid and polyphenol slightly correlated with reducing power including H-RP and L-RP (|*r* | <0.5). This probably related with the specific category and property of flavonoid and polyphenol contained in particular peach [35].

Table 1 suggested that the decay incidence of postharvest peaches had few correlation with total flavonoid content, total polyphenol content, and DRSC, but it had significantly negative correlation with Vitamin C content (*r* = −0.73) and positive correlation with H-RP (*r* = 0.85). Though Vitamin C contributed little to antioxidant activities in terms of DPPH radical scavenging ability and reducing power, it was related with the enhancement to resist decay incidence. Therefore, exogenous CO might restrain the decay incidence through maintaining Vitamin C content. Correlation analysis suggested that firmness was moderately negative correlation with total flavonoid, total polyphenol, and total flavonoid + total polyphenol content, and their correlation coefficients were −0.53, −0.67, and −0.64, respectively; it was high negative correlation with H-DRSC, L-DRSC, and T-DRSC, and the correlation coefficients were −0.53, −0.67, and −0.64, respectively. Moreover, the correlation coefficient between firmness and PAL activity was −0.94. Therefore, CO might maintain the firmness and postpone the shelf life through inducing PAL activity of flesh. This result is similar to Zhang and Li who treated jujube with exogenous CO [16]. With research depth, people found that plant could generate CO. Heme oxygenase oxidation, lipid peroxidation, and ureide metabolism were probably the potential sources of plant CO [36]. Similar to NO and H_2_S, CO takes part in various abiotic stresses and physiological processes such as stomatal movement and lateral root [37]. In plant postharvest physiology, CO was used to protect the green of vegetable and inhibit the browning of lotus root slices. Our results exhibited that exogenous CO might postpone the softening and decay incidence of postharvest peaches, maintain the nutrients and quality, and prolong the shelf life through increasing functional nutrients such as flavonoid, polyphenol, and vitamin C. Without doubt, the exact mechanism should be further investigated in future. In general, oxidative regulation is a dynamic balancing process between systems that produce and scavenge ROS [38]; the initial tissue response to stress leads to ROS production (step I), and this in turn, triggers antioxidant protection systems to ameliorate ROS (step II) but in case the tissue can no longer cope with stress then subcellular or cellular damage occurs (step III). Protection systems mainly include phenolics, ascorbate, glutathione, peroxidase, ascorbate peroxidase, glutathione peroxidase and reductase, and catalase [39]. In particular, there are many mechanisms by which phenolics can act either as antioxidants (as agents for free radical scavenging, hydrogen donation, singlet oxygen quenching, and metal ion chelation) or as substrates for attack by superoxide [40]. Thus, we presumed that CO probably indirectly adjusted the oxidative stress of tissue producing during fruit ripening and senescence process through regulating the flesh polyphenol content, thereby delaying fruit ripening and senescence. The antioxidant defense system induced by CO comprise of ascorbate peroxidase, glutathione reductase, superoxide dismutase, monodehydroascorbate reductase, ascorbic acid reductase, and so on, regulating reactive oxygen species and restraining oxidative stress through the transcription and expression of these enzymes. In our previous study, a similar result about the effect of CO fumigation on the active oxygen metabolism of jujube was acquired (unpublished). Therefore, CO also probably maintained peach quality through regulating active oxygen [41, 42]. As a signal molecule, CO of plant similar to NO might play a physiological role through cGMP pathway. Xuan et al. found that the growth elongation of wheat root induced by CO associated with cGMP signal pathway [43]. Many evidences suggest that CO controls the response process to abiotic stresses via molecular interaction with other signal moleculars, such as NO [44]. Currently, the research about CO in plant postharvest physiology was limited. Since NO as a useful gas to preserve fruit had been widely recognized, whether it participates in CO regulating plant senescence process and whether CO plays a role on plant ripening and senescence via the cGMP pathway need to be further investigated in the future.

## 5. Conclusions

Treating postharvest peaches with CO demonstrated dual effect. In low concentration CO (0.5–10 *μ*mol/L), exogenous CO could delay the decrease of firmness and titrable acid content, restrain the increase of decay incidence, and postpone the variation of soluble solids content, and the positive effect was more obvious with CO concentration enhancement. However, treating peaches with high concentration of CO (20 *μ*mol/L) demonstrated adverse effect. Further researches exhibited that exogenous CO might induce the PAL activity, maintain nutrient contents such as Vitamin C, flavonoid, and polyphenol, and enhance antioxidant activity according to reducing power and DPPH scavenging ability. This result suggested that treating peaches with appropriate concentration of CO was beneficial to quality, nutrients, and antioxidant activity of postharvest peaches during storage period.

## Figures and Tables

**Table 1:** Correlation coefficients between factors related to flesh quality and antioxidant activities in postharvest peaches.

	Firmness	Decay incidence	Total flavonoid	Total polyphenol	Total flavonoid + total polyphenol	Vitamin C	PAL activity	H-DRSC	L-DRSC	T-DRSC	H-RP	L-RP
Decay incidence	−0.75^*^											
Total flavonoid	−0.53	−0.09										
Total polyphenol	−0.67	0.08	0.73^*^									
Total flavonoid + total polyphenol	−0.64	−0.01	0.94^**^	0.92^**^								
Vitamin C	0.29	−0.73^*^	0.65	0.17	0.45							
PAL activity	−0.94^**^	0.67	0.48	0.72^*^	0.64	−0.31						
H-DRSC	−0.83^**^	0.36	0.89^**^	0.72^*^	0.87^**^	0.27	0.74^*^					
L-DRSC	−0.89^**^	0.67	0.30	0.73^*^	0.54	−0.50	0.92^**^	0.58				
T-DRSC	−0.97^**^	0.56	0.70	0.81^*^	0.81^*^	−0.08	0.92^**^	0.91^**^	0.86^**^			
H-RP	−0.48	0.85^**^	−0.48	−0.09	−0.32	−0.96^**^	0.45	−0.04	0.59	0.27		
L-RP	−0.01	−0.22	0.04	0.17	0.11	−0.05	0.00	−0.05	0.22	0.08	0.05	
T-RP	−0.21	0.16	−0.16	0.12	−0.03	−0.44	0.19	−0.06	0.44	0.19	0.46	0.91^**^

^*^ *P* < 0.05, ^**^ *P* < 0.01.
